# Improving synergistic drug combination prediction with signature-based gene expression features in oncology

**DOI:** 10.3389/fphar.2025.1614758

**Published:** 2025-07-17

**Authors:** Mozhgan Mozaffarilegha, Sajjad Gharaghani

**Affiliations:** Laboratory of Bioinformatics and Drug Design (LBD), Institute of Biochemistry and Biophysics, University of Tehran, Tehran, Iran

**Keywords:** drug combination, synergy, drug signature, drug resistance, gene expression

## Abstract

**Background:**

Combination therapies play a crucial role in the treatment of complex diseases, such as cancer. They enhance efficacy, minimize resistance, and reduce toxicity by leveraging synergistic effects. However, identifying effective combinations is challenging due to the vast number of possible pairings and the high-priced costs of experimental validation. Machine learning (ML) and deep learning (DL) models have advanced drug synergy prediction by integrating diverse datasets and modeling the interactions between drugs and cell lines. Despite these advancements, most algorithms primarily rely on drug-specific features, such as chemical structures, with limited incorporation of functional drug information and cellular content features.

**Methods:**

We propose a novel approach that integrates Drug Resistance Signatures (DRS) as a biologically informed representation of drug information. This approach provides a more comprehensive framework for identifying effective combination therapies. We evaluated the predictive power of DRS features across various machine learning models (LASSO, Random Forest, AdaBoost, and XGBoost) and the deep learning model SynergyX. We compared their performance with that of conventional drug signatures and chemical structure-based descriptors.

**Results:**

Our results demonstrate that models incorporating DRS features consistently outperform traditional approaches across all evaluated algorithms. Validation on independent datasets, including ALMANAC, O’Neil, OncologyScreen, and DrugCombDB, confirms the robustness and generalizability of the proposed framework.

**Discussion:**

These findings emphasize the importance of integrating resistance-informed transcriptomic features into computational models. By capturing drug functionality in a biologically relevant context, DRS improves both the accuracy and interpretability of drug synergy prediction, offering a powerful strategy for guiding the discovery of effective combination therapies.

## 1 Introduction

Drug resistance occurs in over 90% of cancer patients, where cancer cells develop tolerance to treatment. Therefore, combination therapy has proven to be an effective method for combating drug resistance (Yardley, 2013). Genetic mutations, epigenetic changes, increased drug efflux, and other complex cellular and molecular mechanisms cause his resistance. Drug resistance can be classified into intrinsic and acquired types based on when it develops (Wang et al., 2023). Intrinsic resistance occurs prior to patient exposure to drugs, which may reduce the drug efficacy from the beginning (Wang et al., 2023; Wang et al., 2019; Holohan et al., 2013). However, acquired resistance develops over time during treatment and is characterized by a decrease in the drug’s effectiveness over time. Acquired resistance can be caused by the activation of a proto-oncogene, which becomes the newly emerging driver gene, mutations, changing expression levels of drug targets, or changes in the tumor microenvironment after therapy. Both intrinsic and acquired resistance are common, with each occurring in roughly 50% of cancer patients who develop drug resistance (Holohan et al., 2013). Therefore, drug combination therapies have become important as promising methods to overcome resistance by simultaneously targeting multiple targets or biological pathways. In addition, the lower dose prescriptions of a single drug can reduce the potential risks of toxicity and side effects.

The increasing availability of large-scale, high-throughput data and drug combination databases has enabled the development of numerous machine learning (ML) and deep learning (DL) computational methods for predicting drug synergy. These methods vary in their representation of biological systems, how they integrate diverse data, and their modeling of the complex interactions between drugs and cellular contexts (Pan et al., 2023; Besharatifard and Vafaee, 2024).

Conventional ML algorithms such as Random Forest (RF), Support Vector Machines (SVM), Gradient Boosting Machines (GBM), K-Nearest Neighbors (KNN), and logistic regression, have been widely used to predict drug combination outcomes (Güvenç et al., 2021; Li et al., 2020). These models typically rely on engineered features such as chemical fingerprints, gene expression profiles, and drug-target interaction data. While they are computationally efficient and relatively interpretable, their capacity to capture nonlinear and higher-order biological interactions is limited. Some ensemble-based variants combine predictions from different feature spaces to enhance robustness and predictive performance (Li et al., 2020; Xia et al., 2018).

The early DL-based methods, such as DeepSynergy (Preuer et al., 2018) and MatchMaker (Kuru et al., 2021), utilize fully connected deep neural networks to learn complex patterns from chemical descriptors and transcriptomic features. To further enhance performance, feature fusion models such as WRFEN-XGBoost (Lu et al., 2021) integrate drug-induced expression perturbations to better model drug interaction effects. Recent developments have introduced multi-view DL models that assign a separate sub-network to each type of input data (such as gene expression, drug structure, or protein abundance), followed by a shared prediction layer. These architectures reduce noise and leverage the complementary strengths of heterogeneous datasets. Models such as BestComboScore (Xia et al., 2018) and DeepDDS (Wang et al., 2022) are key examples of this approach.

Graph convolutional approaches, including DRSPRING (Han et al., 2024) and MFSynDCP (Dong et al., 2024), model drugs, targets, and pathways as interconnected networks. By embedding this structure into the learning framework, these models capture relational patterns that are often missed by flat feature vectors. Similarly, knowledge graph and hypergraph-based methods, such as KGANSynergy (Zhang et al., 2023) and HypergraphSynergy (Liu et al., 2022), are designed to account for higher-order interactions beyond drug pairs. These models are particularly effective in sparse or noisy settings, where they leverage biological priors to infer missing links. Recent graph attention models, like SynergyX (Guo et al., 2024), further prioritize explainability by highlighting the most influential features in synergy prediction.

To overcome the limitations of any single modality or model type, hybrid systems integrate multiple data sources (chemical, genomic, and phenotypic) and learning paradigms (e.g., deep learning, graph neural networks, and ensemble learning). These approaches have demonstrated improved generalizability and predictive stability across diverse datasets. For instance, multi-modal frameworks incorporating drug-pathway-cell line graphs, attention-guided embedding fusion, and pathway-enriched transcriptomic features provide both predictive power and biological insight (Zhang et al., 2023; Peng et al., 2024).

**FIGURE 1:**
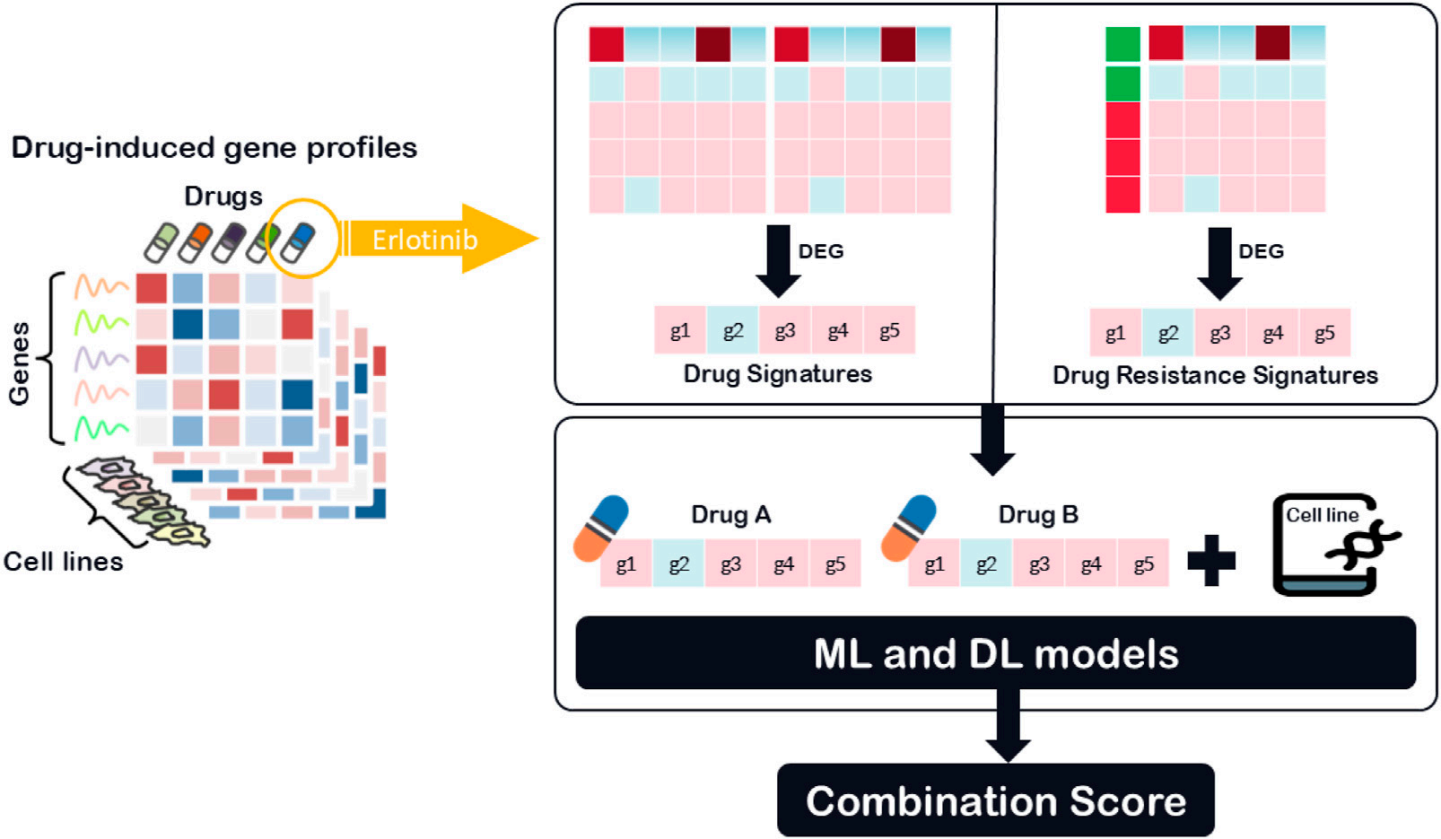
Workflow for predicting drug synergy using transcriptomic signatures features.

While recent hybrid and deep learning models have started to incorporate functional drug data, such as transcriptional profiles and drug-induced gene expression changes, these applications are often limited to general drug signatures or pathway activation scores. Methods such as DeepMDS (Lu et al., 2021) and DRUGSYNC (Zhao and Luo, 2024) utilize transcriptomic features, including drug-induced expression profiles or pathway-level summaries to represent drug function. However, most of these models rely on broad or averaged gene expression data without explicitly modeling resistance-specific transcriptional adaptations. Despite these advances, the detailed integration of drug resistance-specific transcriptional information remains largely unexplored, particularly in the context of drug synergy prediction. In particular, transcriptomic DRS can reveal molecular adaptations that contribute to cancer drug resistance, enabling more accurate predictions of treatment efficacy. To address this gap, To address this gap, we utilize a novel feature class, DRS, which captures transcriptomic changes associated with drug resistance mechanisms, as illustrated in Figure 1. Unlike traditional models that rely primarily on chemical structures or general drug-induced transcriptional responses, DRS features provide a functional perspective by highlighting gene expression differences between drug-sensitive and drug-resistant cancer cell lines. To evaluate the generalizability and effectiveness of this feature type, we analyzed its performance across various modeling strategies. These included four classical machine learning algorithms: LASSO, Random Forest, AdaBoost, and XGBoost, as well as the deep learning framework SynergyX. Our findings suggest that incorporating functional drug data, particularly resistance-related signatures, substantially improves predictive performance in drug combination modeling.

## 2 Materials and methods

### 2.1 Datasets

The Drug Combination Database aggregates experimental data on drug pair interactions, including synergy scores derived from *in vitro* assays conducted across various cell lines. We utilized five datasets: DrugComb (Zheng et al., 2021), O’Neil (O’Neil et al., 2016), Oncology Screen (O’Neil et al., 2016), DrugCombDB (Liu et al., 2020), and Alamanac (Holbeck et al., 2017) for benchmarking predictive models against experimentally validated drug combinations. Among them, DrugComb is the primary dataset in this study, and it included 739,964 drug combination experiments and introduces a novel synergy metric, the S score (Malyutina et al., 2019). This metric quantifies drug synergy by measuring the disparity between the dose-response curves of a drug combination and its constituent single agents. Table 1 summarizes the drug combination synergy data in different datasets with various synergy types.

**TABLE 1:** Overview of datasets utilized for drug synergy prediction analysis.

Datasets	Drugs	Cell lines	Combination
DrugComb	354	170	330917
DrugCombDB	600	68	60932
O’Neil	38	39	23062
Oncology Screen	21	29	4176
Alamanac	118	118	296503

### 2.2 Drug signature features

The LINCS database provided extensive gene expression data from diverse cell lines exposed to various drugs (Subramanian et al., 2017). Its large-scale repository included transcriptomic signatures across various experimental conditions, such as different drug concentrations and time points. We used LINCS data 24 h after treatment with 10 μM drug concentration, as it was the most common condition in the LINCS dataset.

We also obtained drug response metrics for a wide array of cancer cell lines from the GDSC database (Iorio et al., 2016), including IC_50_ values and dose-response curves for several thousand anticancer agents.

We extracted Level 5 transcriptomic signatures from the LINCS database and associated these profiles with cell viability data from the GDSC to analyze drug-induced responses across multiple cell lines. The integration of LINCS and GDSC datasets involved identifying overlapping drugs by cross-referencing drug identifiers and filtering for matches, resulting in a final set of common drugs.

To characterize drug sensitivity and resistance, cell lines have been grouped from the GDSC based on their IC_50_ values, using the median IC_50_ across all cell lines as a threshold, following established methodologies (Wang et al., 2019). We define the sensitivity status $S_{i}$ of the i cell line as:

$$S_{i}=\left\{\begin{array}{l} if\;{IC}_{50}^{i}<median\left({IC}_{50}^{i}\right)\;sensitive\;\\ if\;{IC}_{50}^{i}\geq median\left({IC}_{50}^{i}\right)\;resistance \end{array}\right.$$

Where ${IC}_{50}^{i}$ denotes the IC_50_ value of cell line j for a given drug.

Differential gene expression analysis was performed using two different approaches: Conventional Drug Signature (DS): This signature compares gene expression between treated and untreated conditions for a given drug across a fixed cell line. For each gene i , the differential expression score is computed as:

$$\mu_{i}^{S}-\mu_{i}^{R}={∆}_{i}^{SR}$$

Where:

$${\;\mu }_{i}^{R}=\mu \sum_{j\epsilon T}\frac{1}{\left|T\right|}{\;\mu }_{i}^{S}=\mu \sum_{j\epsilon U}\frac{1}{\left|U\right|}$$

Let T be the set of samples treated with a specific drug, U be the set of control (untreated) samples. For each gene i , the mean expression level under treated $\mu_{i}^{R}$ and control $\mu_{i}^{S}$ conditions are calculated by averaging the normalized expression values across all samples in each group. The differential expression score ${∆}_{i}^{SR}$ is then defined as the difference between these two means. A statistical test (e.g., t-test or moderated t-statistic) is used to compute the significance ( p−value ) for each ${∆}_{i}^{SR}$ and a threshold on adjusted p−values is used to identify significantly up/downregulated genes.

Drug Resistance Signature: This signature compares gene expression between resistant and sensitive cell lines in response to the same drug.

$$\mu_{i}^{R}-\mu_{i}^{S}={∆}_{i}^{SRD}$$

For each gene i , the mean expression in resistance $\mu_{i}^{R}$ and sensitive $\mu_{i}^{S}$ samples are computed. The resistance-associated differential expression score ${∆}_{i}^{SRD}$ reflects the gene expression changes between resistance and sensitive contexts. These scores represent resistance-specific transcriptomic patterns that serve as functional drug features in our modeling framework.

### 2.3 Comparative analysis of models

To evaluate the predictive value of different drug signature representations, we conducted a comparative analysis using four widely adopted machine learning algorithms—LASSO, AdaBoost, Random Forest (RF), and XGBoost—as well as the deep learning model SynergyX, a recent attention-guided multi-modal model. This evaluation aimed to assess how effectively each model leverages structural features, DS, and DRS to predict drug combination synergy.

All models were trained and evaluated on the same datasets under identical conditions, utilizing the same input features, model architectures, and training parameters as specified in their respective original studies. This standardized approach ensured a fair and unbiased comparison of performance across methods. In this study, SynergyX is utilized in the main model to predict drug synergy by leveraging functional data, such as drug resistance signatures. Built on a multi-modal architecture, it integrates diverse feature spaces, including drug features, gene expression, and functional cell-level data. A key component, its Cross-Modal Fusion Encoder, captures complex interactions between different data modalities, such as molecular properties and cellular response features (Guo et al., 2024).

### 2.4 Model evaluation

We employed a stratified 5-fold cross-validation strategy to evaluate the performance of all models. This method ensured that the distribution of the target variable (synergy scores) was preserved across training and testing splits, reducing the potential for data imbalance to affect model performance. Each experiment was repeated ten times with different random seeds, ensuring that the results were robust and not sensitive to initialization or sampling variability.

We used Mean Squared Error (MSE), Root Mean Squared Error (RMSE), R-squared (R^2^), and correlation as evaluation metrics for the regression prediction task. These metrics provided a comprehensive assessment of the models’ predictive accuracy, ability to capture variance, and rank-order relationships between predicted and observed synergy scores. Additionally, 95% confidence intervals (CIs) were computed for each metric to assess the reliability and variability of the results.

To further validate the generalizability and robustness of our approach, we tested all models on four independent benchmark datasets: ALMANAC, DrugCombDB, Oncology Screen, and O’Neil’s dataset. This evaluation aimed to demonstrate the model’s ability to perform well on unseen datasets and across diverse experimental conditions, a critical requirement for real-world applications in predicting drug synergy.

## 3 Results

### 3.1 Evaluation of model performance across classes

The following analysis compares the predictive performance of various machine learning and deep learning models across three distinct feature categories: structural drug descriptors, DS, and DRS. As shown in Table 2, the SynergyX model trained with DRS features consistently achieves the lowest Mean Squared Error (MSE) and Root Mean Squared Error (RMSE), outperforming traditional models such as LASSO, Random Forest (RF), AdaBoost, and XGBoost (XGB). Notably, in the DRS feature category, SynergyX achieves the best performance with an MSE of 92.16 ± 1.82, significantly better than other models.

**TABLE 2:** Comparative performance of machine learning models across three feature categories: structure, drug resistance (DR) and DRS.

Feature Category	Measure	LASSO	AdaBoost	RF	XGB	SynergyX
Structure	MSE	331.40 ± 5.40	346.22 ± 4.99	**163.58 ± 1.82**	147.15 ± 1.19	92.81 ± 1.29
	95%Cl	[316.39 346.40]	[332.38 360.07]	[158.51 168.64]	[143.86 150.45]	[89.23 96.38]
	P-value	4.24E-07	2.58E-07	9.29E-08	2.55E-08	2.22E-07
DS	MSE	345.83 ± 4.86	351.57 ± 5.16	346.16 ± 4.85	346.15 ± 4.79	106.18 ± 1.90
	95%Cl	[332.34 359.33]	[337.25 365.88]	[332.70 359.61]	[332.85 359.45]	[100.92 111.45]
	P-value	2.34E-07	2.34E-07	2.34E-07	2.34E-07	6.10E-07
DRS	MSE	**324.59 ± 3.99**	**341.57 ± 4.89**	165.65 ± 2.07	**144.51 ± 3.09**	**92.16 ± 1.82**
	95%Cl	[313.51 335.67]	[327.99 355.15]	[159.91 171.40]	[135.93 153.09]	[87.10 97.22]
	P-value	1.37E-07	2.52E-07	1.46E-07	1.25E-06	9.15E-07

Bold values indicate the best performance (i.e., lowest error or highest correlation) among the compared models within each feature category.

In addition to accuracy metrics, the Pearson correlation coefficients of greater than 0.70 for most models in the DRS category indicate that drug resistance signatures capture biologically relevant patterns in synergy prediction more effectively than drug signatures (0.72) and structural features (0.68).

**FIGURE 2:**
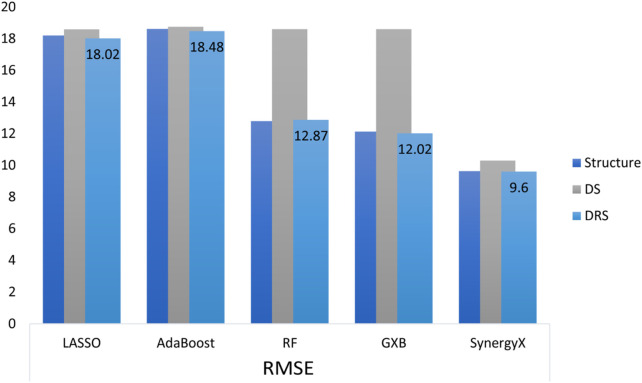
Comparison of RMSE across different machine learning and deep learning models utilizing three feature categories: Structure, Drug Signature (DS), and DRS.

Similarly, Spearman correlations were more consistent in the DRS class, with values remaining above 0.80. This result indicates that functional drug-response data not only improves predictive accuracy but also enhances the model’s ability to effectively capture rank-order relationships between drug combinations. The DRS feature class exhibited narrower confidence intervals (CIs) for both the mean squared error (MSE) and root mean squared error (RMSE) metrics, indicating higher model stability and reliability. For example, the 95% CI for RMSE in the DRS class 9.73 ± 0.10 is significantly tighter than that of the structural feature class, as shown in Figure 2, indicating reduced variability and more consistent predictions.

The R^2^ metric, a crucial measure of predictive accuracy, further highlights the importance of DRS features. In the structural feature class, the highest R^2^ value of 0.67 ± 0.07 was achieved by SynergyX, indicating moderate predictive performance. Within the drug signature (DS) class, SynergyX again outperformed other models with an R^2^ of 0.71 ± 0.04, compared to 0.61 ± 0.03 for XGBoost (XGB) and 0.55 ± 0.03 for Random Forest (RF). The DRS feature class demonstrated the highest R^2^ values, highlighting the superior predictive capability of functional features. This strong R^2^ score reinforces the importance of functional drug-response data in accurately modeling complex drug interactions.

Additionally, The AUC (Area Under the Curve) metric, used to assess model classification power in synergy prediction, further supports the superior performance of DRS features. Although this is a regression study, a commonly used synergy threshold of 10 was applied for classification purposes. SynergyX achieved the highest AUC in the DRS class at 0.74 ± 0.01, followed by XGBoost (XGB) at 0.72 ± 0.01, while traditional models like AdaBoost and Random Forest (RF) scored lower at 0.69 ± 0.00 and 0.70 ± 0.01, respectively. Results from the drug signature (DS) and Structure classes revealed performance limitations when using less detailed features, with a peak AUC of 0.72 ± 0.01 for SynergyX in the DS class and 0.74 ± 0.01 in the Structure class. While structure-based features performed well for classification tasks, their regression performance was less consistent. In contrast, DRS features not only improved regression accuracy but also enhanced classification robustness and interpretability.

To further evaluate the effectiveness of the DRS, we applied the SynergyX model to four widely used benchmark datasets: ALMANAC, DrugCombDB, OncologyScreen, and O’Neil. As summarized in Table 3, we compared the predictive performance of models trained with DRS versus those trained with DS across multiple evaluation metrics.

**TABLE 3:** Performance comparison of SynergyX using Drug Signature (DS) and DRS features across four benchmark datasets.

Models		MSE	RMSE	R2	Pearson	Spearman
Alamanac	DR	3,219.46	56.74	0.02	0.15	0.17
	DRS	**1,273.28**	**35.68**	**0.61**	**0.78**	**0.73**
DrugCombDB	DR	25.73	5.07	0.06	0.25	0.27
	DRS	**10.80**	**3.28**	**0.60**	**0.78**	**0.86**
OncologyScreen	DR	530.33	23.02	0.07	0.28	0.27
	DRS	**230.83**	**15.19**	**0.59**	**0.80**	**0.76**
Oneils	DR	428.70	20.70	0.02	0.19	0.18
	DRS	**163.35**	**12.78**	**0.62**	**0.79**	**0.79**

Bold values indicate the best performance (i.e., lowest error or highest correlation) among the compared models within each feature category.

In the ALMANAC dataset, the DRS-based model achieved substantially lower error rates (MSE: 1,273.28, RMSE: 35.68) and markedly higher correlation scores (Pearson: 0.78; Spearman: 0.73) than the DS-based model, which showed weak correlations (Pearson: 0.15; Spearman: 0.17) despite reporting a marginally higher R^2^.

In DrugCombDB, while the DS model yielded slightly lower MSE and RMSE, the DRS-based model demonstrated significantly superior rank-order consistency, with a Spearman correlation of 0.78 compared to 0.25 for the DS model. For the OncologyScreen dataset, DRS again outperformed DS, achieving better error metrics (MSE: 230.83; RMSE: 15.19) and higher correlations (Pearson: 0.80; Spearman: 0.76), while DS showed weaker predictive performance (MSE: 530.34; Pearson: 0.28; Spearman: 0.27).

Similarly, in O’Neil’s dataset, the DRS-based model outperformed DS across all metrics, achieving an MSE of 163.35, RMSE of 12.78, and strong correlation values (Pearson and Spearman: 0.79), indicating robust predictive accuracy and rank-order reliability.

### 3.2 Comparative analysis between drug and drug resistance signatures

**FIGURE 3:**
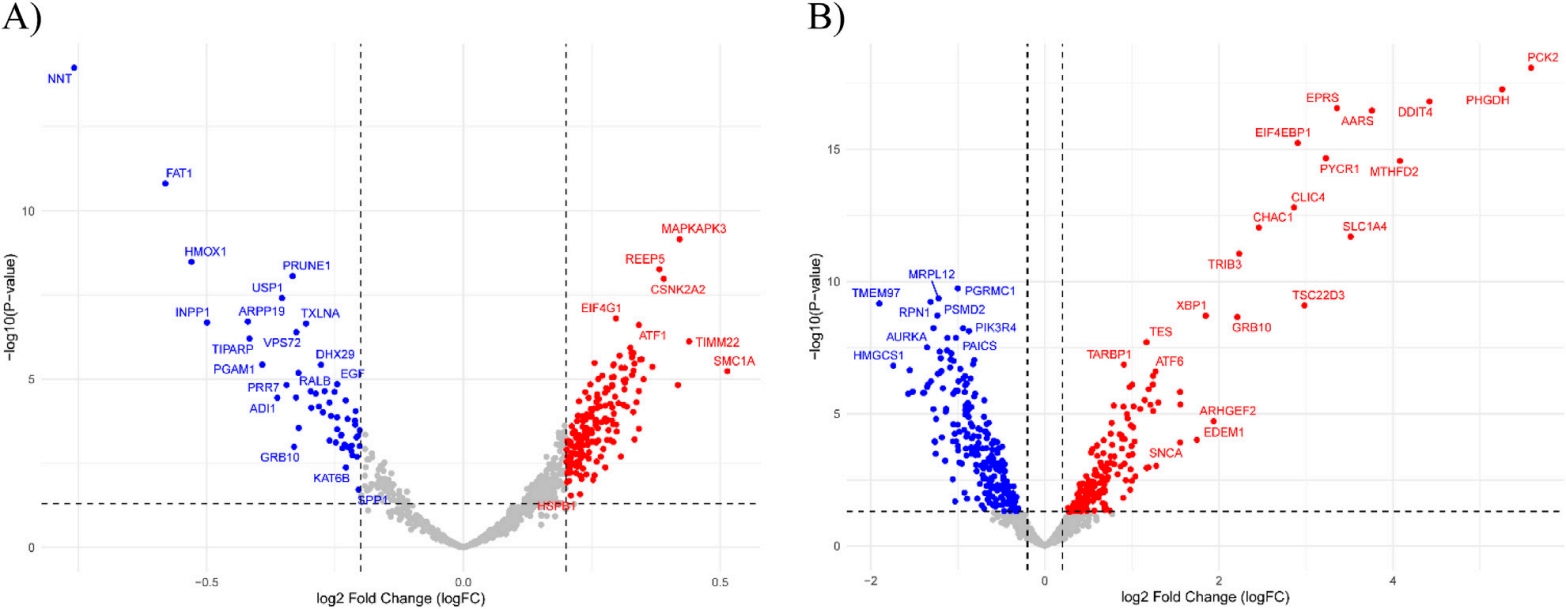
Volcano plots of differential gene expression analysis. **(A)** DS highlights general drug-induced expression changes in response to Erlotinib. Significantly upregulated genes include MAPKAPK3, CSNK2A2, and EIF4G1, associated with cell cycle regulation, signal transduction, and translation initiation. **(B)** DRS displays a distinct expression profile with significant upregulation of resistance-associated genes, including EIF4EBP1, TRIB3, and SLC1A4, which are linked to EGFR signaling modulation and metabolic adaptation. Dashed lines indicate the thresholds for log2 Fold Change (logFC) and −log10 (P-value).

The gene expression profiles related to Erlotinib in both the DS and DRS are shown in Figure 3. The volcano plot for the DS (Figure 3A) reveals significant upregulation of genes like MAPKAPK3, CSNK2A2, and EIF4G1, which are involved in cell cycle regulation, signal transduction, and translation initiation. These genes suggest enhanced cellular adaptability and survival mechanisms in response to Erlotinib treatment. Downregulated genes such as GRB10 and FAT1 are linked to growth signaling and cell adhesion, indicating potential inhibition of survival pathways commonly associated with EGFR signaling. The broader gene distribution in the DS suggests a less specific but more comprehensive representation of drug response, capturing both direct and indirect effects of Erlotinib exposure.

In contrast, the DRS (Figure 3B) focuses on a more refined set of genes directly tied to resistance mechanisms and Erlotinib’s targeted pathways. Upregulated genes, including EIF4EBP1, TRIB3, and SLC1A4, are associated with modulation of EGFR signaling, stress response, and metabolic adaptation, emphasizing their direct role in driving resistance. Additionally, the downregulation of genes such as XBP1 and TSC22D3, involved in stress response and apoptotic regulation, highlights altered cellular pathways that reduce sensitivity to Erlotinib. This refined gene expression pattern underscores key mechanisms that contribute to the development of drug resistance.

To elucidate the molecular pathways driving Erlotinib’s therapeutic effects and resistance mechanisms, we performed pathway enrichment analysis on gene expression profiles derived from DS and DRS analyses. This approach allowed us to identify distinct biological processes associated with Erlotinib sensitivity and acquired resistance.

**FIGURE 4:**
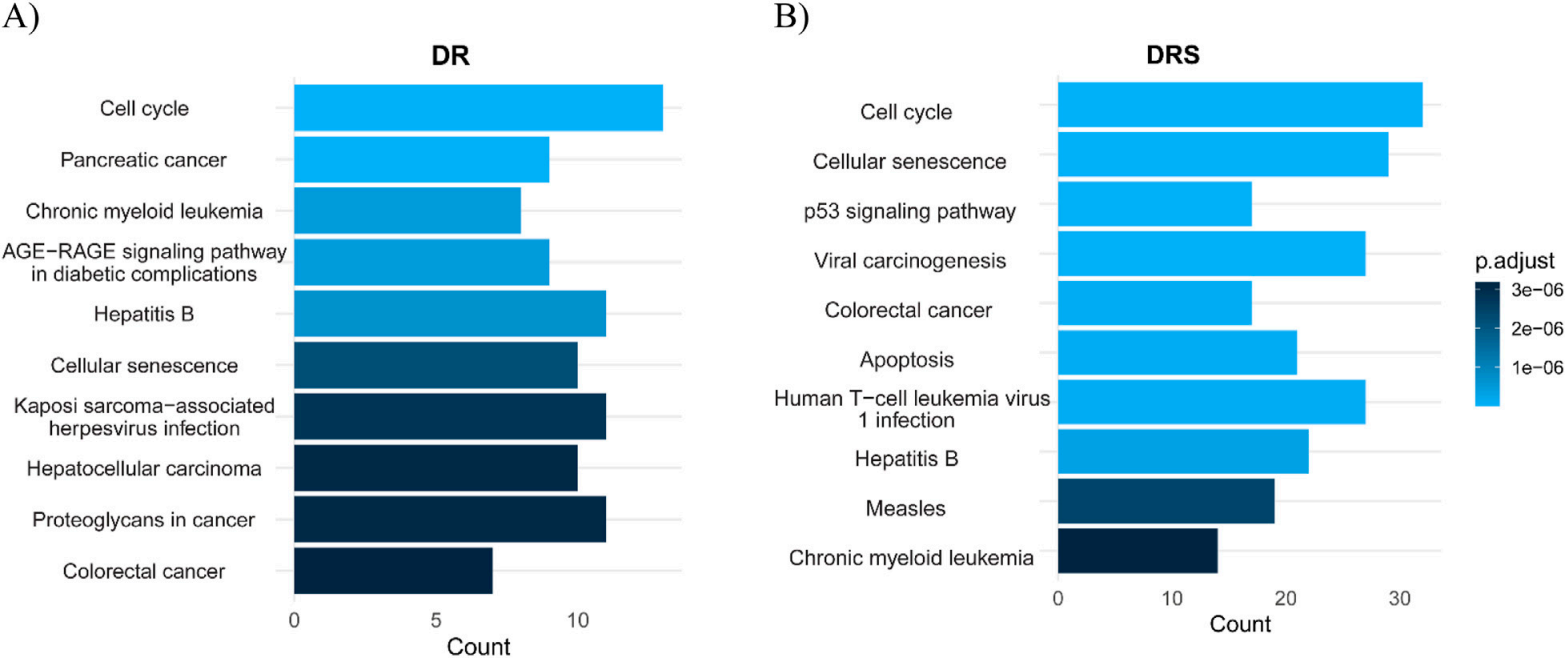
Pathway enrichment analysis comparing DRS and Drug Signature (DS) for Erlotinib. **(A)** DS highlights pathways associated with cancer progression and tumor signaling. These pathways suggest Erlotinib’s impact on tumor biology and its potential involvement in regulating cancer-associated processes. **(B)** DRS shows pathways mainly related to cell cycle control, p53 signaling, cellular senescence, and apoptosis, underscoring mechanisms of resistance and cellular survival. The x-axis represents either pathway count or adjusted p-values (−log10), reflecting the statistical significance of pathway enrichment.

The results, presented in Figure 4, show the most significantly enriched pathways based on adjusted p-values (on a-log10 scale). In the DS analysis (Figure 4A), the most enriched pathways included Colorectal cancer, Proteoglycans in cancer, Hepatocellular carcinoma, and Kaposi sarcoma-associated herpesvirus infection. Notably, pathways such as Chronic myeloid leukemia, Pancreatic cancer, and Cell cycle regulation also show significant enrichment. These pathways are consistent with Erlotinib’s known mechanism of action as an EGFR inhibitor, influencing cancer proliferation, senescence, and stress-response pathways, which likely contribute to Erlotinib sensitivity.

In contrast, the DRS analysis (Figure 4B) revealed a different enrichment profile. Pathways such as Apoptosis, Colorectal cancer, Viral carcinogenesis, p53 signaling pathway, Cellular senescence, and Cell cycle are among the top enriched pathways. These findings suggest a prominent role of genomic stability and cell survival mechanisms in resistance. The upregulation of DNA damage response and cell cycle regulation pathways indicates that Erlotinib-resistant cells may activate compensatory mechanisms to enhance their survival and promote resistance. These results highlight the distinct biological processes involved in Erlotinib sensitivity versus resistance. While sensitive cells show enrichment in cancer-related and stress response pathways, resistant cells exhibit pathways associated with survival, apoptosis, and immune-related processes related to viral infections, potentially facilitating their adaptation and resistance to treatment.

### 3.3 Drug synergy predictions based on drug resistance signature

To further evaluate the performance of our model, we assessed its ability to identify novel and biologically meaningful drug combinations. For this purpose, we selected a set of 68 FDA-approved anticancer drugs commonly used in breast cancer treatment. To capture the influence of cellular context on drug synergy, we selected two biologically distinct yet estrogen receptor-positive (ER+) human breast cancer cell lines: MCF7 and T47D. This selection enabled us to evaluate both the consistency of predicted drug combinations across different cellular environments and the discriminative power of the proposed feature space. The chosen drugs were identified based on their overlap within the LINCS and GDSC databases, ensuring compatibility for downstream analyses. We then generated all possible drug pairs and employed the SynergyX deep learning model, enhanced with DRS features, to predict synergy scores. This framework enabled a robust, context-specific evaluation of model performance and biological relevance.

The top-ranking pairs for each cell line are presented in Tables 4, 5, emphasizing the influence of cell line specificity on the predicted results. Table 4 displays the top 5 predicted drug combinations for the MCF7 cell line. These findings indicate a potential role for Methotrexate in mediating synergistic interactions that are specific to the MCF7 cell line. In contrast, Table 5 presents the top five predicted drug combinations for the T47D cell line, where Anastrozole-based combinations consistently achieved the highest synergy scores—particularly Anastrozole in combination with Methotrexate or Lapatinib. Notably, predicted synergy scores were consistently higher in T47D than in MCF7, emphasizing the importance of cell line-specific biological context in influencing combination outcomes. These results demonstrate that drug synergy predictions are highly dependent on the underlying cellular background, with the T47D model exhibiting generally stronger synergistic responses. This variation highlights the significant impact of factors such as genetic profiles, molecular signaling networks, and baseline resistance phenotypes on influencing drug-drug interactions.

**TABLE 4:** Top 5 Predicted Synergistic Drug Combinations for the MCF7 Cell line.

Rank	Drug A	Drug B	Pred-Score
1	Anastrozole	Methotrexate	12.59
2	Cyclophosphamide	Methotrexate	9.91
3	Letrozole	Methotrexate	8.25
4	Cyclophosphamide	Lapatinib	7.42
5	Anastrozole	Lapatinib	7.09

**TABLE 5:** Top 5 Predicted Synergistic Drug Combinations for the T47D Cell line.

Rank	Drug A	Drug B	Pred-Score
1	Anastrozole	Methotrexate	23.87
2	Anastrozole	Lapatinib	17.41
3	Cyclophosphamide	Methotrexate	14.60
4	Letrozole	Methotrexate	13.38
5	Cyclophosphamide	Lapatinib	12.83

Among the top-ranked drug pairs, combinations involving Anastrozole and Methotrexate consistently emerged across both MCF7 and T47D cell lines, suggesting their potential as robust synergistic partners. Furthermore, T47D-specific combinations such as Anastrozole–Lapatinib and Letrozole–Olaparib represent promising candidates for novel combination therapies.

The observed synergy between Anastrozole, an aromatase inhibitor, and Methotrexate, a dihydrofolate reductase inhibitor, is likely driven by their complementary mechanisms of action. Anastrozole suppresses estrogen production, thereby inhibiting the growth of estrogen receptor (ER)-positive breast cancer cells. In parallel, Methotrexate impairs DNA synthesis, enhancing cytotoxic effects in rapidly proliferating tumor cells.

Importantly, the ability of DRS to accurately predict this synergy underscores their strength in capturing adaptive cellular responses, particularly how tumor cells reprogram their survival pathways when exposed to dual-targeting strategies. This finding highlights the practical value of DRS-informed models in identifying drug combinations that exploit functional vulnerabilities in resistant cancer phenotypes.

## 4 Discussion

This study demonstrates the effectiveness of DRS features in improving the prediction of synergistic drug combinations by incorporating functional transcriptomic responses to drug treatment. Unlike conventional models that rely on chemical structures or general gene expression data, DRS-guided models provide more mechanistic insight into drug interactions, identifying combinations that either target complementary biological processes or performance on the same resistance pathway.

To evaluate the biological relevance of the DRS-based feature, we conducted a case study using Erlotinib, a selective EGFR (epidermal growth factor receptor) inhibitor. Comparing general drug signature profiling with DRS analysis revealed key differences in the pathways associated with Erlotinib resistance (Harada et al., 2012; Kanda et al., 2013; Liao et al., 2020; Jakobsen et al., 2017). While the DS approach identified a broad range of pathways, including cellular stress responses and metabolic adaptations, the DRS approach provided more specific mechanistic insights, directly linking resistance to compensatory survival mechanisms. Both profiling methods confirmed EGFR signaling as central to Erlotinib’s function. However, DRS uniquely identified adaptive resistance pathways, such as the PI3K-Akt and p53 signaling pathways, which promote cellular survival and proliferation despite EGFR inhibition. These pathways were significantly enriched in resistant profiles, suggesting that resistant cells leverage compensatory signaling networks to bypass the inhibitory effects of Erlotinib (Zhou et al., 2021; He et al., 2021).

Furthermore, DRS features identified specific upregulated genes associated with Erlotinib resistance, including EF4BP1, TRIB3, and SLC1A4, which are known to drive alternative survival pathways (Wan et al., 2020). These findings suggest that targeting compensatory signaling pathways, such as the PI3K/Akt pathway, may enhance the efficacy of Erlotinib when used in combination therapies. Conversely, downregulated genes, such as XBP1 and TSC22D3, which are involved in oxidative stress regulation and apoptosis, indicate a reduced apoptotic response in resistant cells, further reinforcing the importance of functional resistance profiling. These findings suggest the importance of incorporating DRS-based profiling into resistance studies, as it offers mechanistic clarity beyond general drug response signatures. The identification of resistance-associated pathways, particularly the PI3K/Akt signaling pathway, presents potential therapeutic targets. Targeting these compensatory survival pathways in combination with Erlotinib may enhance its efficacy and help overcome resistance. Overall, DRS analysis offers a refined framework for understanding acquired resistance mechanisms and informs the rational design of combination therapies aimed at improving outcomes in EGFR-targeted treatments.

The top-ranked combination of Anastrozole and Methotrexate exemplifies how DRS features can identify drug interactions based on complementary mechanisms of action. Anastrozole, an aromatase inhibitor, reduces estrogen receptor (ER)-positive breast cancer growth by suppressing estrogen synthesis, thereby limiting tumor proliferation (Milani et al., 2009). Methotrexate, a dihydrofolate reductase inhibitor, disrupts nucleotide synthesis, leading to impaired DNA replication and enhanced cytotoxicity (Jolivet et al., 1983). This synergy highlights how hormonal signaling inhibition and nucleotide depletion can work in concert to enhance therapeutic efficacy, a pattern effectively captured by DRS-based predictive models. The ability of DRS-based models to predict this synergy suggests that transcriptomic resistance signatures effectively capture adaptive survival responses in tumor cells, enabling the identification of functionally relevant drug interactions that may be ignored by traditional structure-based models (Ma et al., 2019). DRS-guided models also prioritize synergistic drug pairs that target the same resistance pathway, as demonstrated by the synergy between Cyclophosphamide and Methotrexate. Cyclophosphamide, an alkylating agent, induces DNA crosslinking and replication stress, leading to genomic instability. Methotrexate, by depleting nucleotide pools, further exacerbates the accumulation of DNA damage, leading to heightened cytotoxic effects and cell death (Sahrayi et al., 2021).

Conventional synergy prediction models, which primarily rely on chemical properties or generalized transcriptional profiles, often lack the resolution needed to identify pathway-specific interactions. As a result, they may overlook critical mechanistic synergies that arise from functional adaptations within resistant cancer cells. This limitation leads to an incomplete understanding of compensatory survival pathways, thereby restricting the ability of predictive models to accurately identify effective drug combinations. By integrating DRS features, our model addresses these challenges by effectively identifying functional synergies that exploit shared resistance mechanisms, thereby providing a more precise and biologically relevant framework for predicting drug synergy.

While this study demonstrates the effectiveness of DRS in enhancing the prediction of drug synergy, several limitations should be considered. Although comprehensive validation using experimental assays would enhance the confidence and translational relevance of the identified drug combinations, our study relied exclusively on large-scale, well-curated datasets for model training and evaluation. Additionally, the dependence on the LINCS and GDSC databases introduces coverage limitations and potential bias due to the incomplete overlap of drugs, cell lines, and treatment conditions. Another limitation lies in deriving resistance signatures from a single post-treatment time point (24 h), which may not adequately capture the temporal complexity and dynamic evolution of drug resistance.

In future work, we aim to integrate single-cell transcriptomics, consider multi-time-point resistance profiling, and develop multi-modal models that incorporate genomic and phenotypic context to improve biological fidelity and clinical relevance.

## 5 Conclusion

This study highlights the importance of incorporating drug resistance-specific functional data in predicting synergistic drug combinations, demonstrating that DRS features enhance predictive accuracy by capturing adaptive transcriptomic responses to therapy. By systematically comparing DRS to structural and general drug signature features across multiple machine learning and deep learning models, SynergyX, we demonstrated that DRS consistently outperforms other feature types in terms of predictive accuracy, rank-order stability, and interpretability. Despite certain limitations, such as reliance on pre-existing datasets and absence of experimental validation, the proposed framework provides a scalable and mechanistically insightful approach for prioritizing effective drug combinations. These findings pave the way for future efforts to integrate multi-omic, temporal, and single-cell data into resistance-aware synergy prediction models, ultimately guiding the development of more precise and personalized combination therapies in oncology.

## Data Availability

Publicly available datasets were analyzed in this study. This data can be found here: https://github.com/mozaffarilegha/DrugCombinationPredicrion_DRS.
